# Learning analytics in virtual laboratories: a systematic literature review of empirical research

**DOI:** 10.1186/s40561-023-00244-y

**Published:** 2023-03-09

**Authors:** Ramy Elmoazen, Mohammed Saqr, Mohammad Khalil, Barbara Wasson

**Affiliations:** 1 School of Computing, University of Eastern Finland, Yliopistokatu 2, 80100 Joensuu, Finland; 2 Centre for the Science of Learning and Technology (SLATE), University of Bergen, Bergen, Norway

**Keywords:** Virtual laboratory, Remote laboratories, Learning analytics, Distance education, Online learning

## Abstract

Remote learning has advanced from the theoretical to the practical sciences with the advent of virtual labs. Although virtual labs allow students to conduct their experiments remotely, it is a challenge to evaluate student progress and collaboration using learning analytics. So far, a study that systematically synthesizes the status of research on virtual laboratories and learning analytics does not exist, which is a gap our study aimed to fill. This study aimed to synthesize the empirical research on learning analytics in virtual labs by conducting a systematic review. We reviewed 21 articles that were published between 2015 and 2021. The results of the study showed that 48% of studies were conducted in higher education, with the main focus on the medical field. There is a wide range of virtual lab platforms, and most of the learning analytics used in the reviewed articles were derived from student log files for students’ actions. Learning analytics was utilized to measure the performance, activities, perception, and behavior of students in virtual labs. The studies cover a wide variety of research domains, platforms, and analytical approaches. Therefore, the landscape of platforms and applications is fragmented, small-scale, and exploratory, and has thus far not tapped into the potential of learning analytics to support learning and teaching. Therefore, educators may need to find common standards, protocols, or platforms to build on each others’ findings and advance our knowledge.

## Introduction

The COVID-19 coronavirus pandemic has created an extremely difficult situation that causes anxiety in the academic field. Practical sessions and experiments in schools and universities have been suspended, which are essential for students’ experience and skill development in laboratory-based disciplines (Vasiliadou, 2020). Despite the pandemic conditions, some specialties have started to use virtual labs for teaching biology, chemistry, and the natural sciences. Virtual labs have the advantages of unlimited time, immediate feedback, experiment repetition, and safety for students and the subjects of the experiment (Vasiliadou, 2020). Students’ experience with virtual and simulated experiments helps prepare them for their physical laboratories and offers a reasonable solution—at least in emergencies—(Breakey et al., 2008). Technology affords students several means of communication, allowing students to interact with teachers, ask for help, or provide feedback about their learning. Furthermore, students can conduct virtual experiments in groups, allowing for social engagement and collaboration through teamwork (Manchikanti et al., 2017). Virtual laboratories can generate digital traces to monitor students' learning and identify their learning strategies. These traces of students' interactions with virtual labs revealed an enhancement in students' ability to solve problems, engage in critical thinking, develop laboratory skills, and acquire knowledge (Ramadahan & Irwanto, 2018). To take advantage of such data, the "learning analytics" field was conceptualized to provide insights into learning by analyzing various student-generated data (Hantoobi et al., 2021).

Learning analytics (LA) is commonly defined as “the measurement, collection, analysis, and reporting of data about learners, learning environments, and contexts to understand and optimize learning and their environments” (SoLAR, 2011). Therefore, LA adopts a data-driven strategy in educational settings with the ultimate goal of enhancing and optimizing the educational experience for students and teachers. LA has a broad range of applications in many fields of education, from preschool to postgraduate studies (Adejo & Connolly, 2017). The LA implementation may provide educational institutions and stakeholders with multiple significant benefits. (Howell et al., 2018; Ifenthaler, 2017). These include LA being used for students’ collaboration measurement (Saqr, Elmoazen, et al., 2022), grade prediction (Agudo-Peregrina et al., 2014; Strang, 2017), learning gap identification (Nyland et al., 2017), failure prediction (Tempelaar et al., 2018), decision making (Vanessa Niet et al., 2016), active learning support (Kwong et al., 2017), profiling students (Khalil & Ebner, 2017) and assessment improvement (Azevedo et al., 2019).

LA has been implemented in many contexts, such as the early identification of at-risk students for underachievement, the tracking of students' online activity, the provision of automated feedback, the facilitation of learning strategies, and the optimization of teamwork in collaborative learning (Kaliisa et al., 2022; Papamitsiou & Economides, 2014). Previous systematic reviews have either narrowed in on the technology and design of virtual laboratories in a single discipline, such as biology (Udin et al., 2020) or chemistry (P. ), covered a wider range of disciplines while focusing on a single technology, such as virtual reality (Rahman et al., 2022) or provided a more broad-based review of the theoretical and practical approaches of virtual labs in various fields (Reeves & Crippen, 2021). However, a systematic review that synthesizes research about how learning analytics are used to monitor, support, or assess virtual laboratory work does not exist. In this study, we aim to bridge such a gap and contribute to the literature with a systematic review encompassing all research about learning analytics and virtual laboratories. We investigate the characteristics, research methods, and findings of learning analytics in virtual labs. Therefore, the main research questions for this study are: How has research on virtual laboratories used learning analytics in regards to educational levels, subjects, applications, and methods of analysis?

## Background

### Virtual labs

Technology-based training is growing across many areas of practice, and education is not an exception. Organizations are adopting virtual and simulated applications to improve trainees’ working skills, problem-solving strategies, and self-directedness (Dalgarno et al., 2003; Richard et al., 2006). Virtual laboratories offer the opportunity to practice several times, anytime, at any pace. Most importantly, they offer safe practice without fear of harm to themselves, equipment, or subjects. Virtual labs have provided students with access to large equipment such as telescopes (Slater et al., 2014), expensive devices such as electron microscopes (Childers & Jones, 2015), risky techniques such as radioactivity measurements (Jona & Vondracek, 2013), and biotic interactions such as cell stimulation (Hossain et al., 2016). Students can access virtual labs via computers and mobile devices, providing a new dimension for students (Lynch & Ghergulescu, 2017). Virtual labs range from simple 2D video games to interactive 3D simulations that provide a more engaging learning environment. Some provide students with instructions and technical directions to complete difficult tasks, whereas others are open-ended (Jones, 2018). Virtual labs have many advantages compared to traditional labs, including less cost, easy access, time-saving, environmental safety, and adaptability (Ali & Ullah, 2020). However, one of the possible drawbacks of virtual labs is that, unlike conventional labs, they do not always offer the same learning environment or the same opportunities for student interactions (Lynch & Ghergulescu, 2017).

Various organizations have created a variety of virtual laboratories, with many of them available as open-source software. The Go-Lab and LiLa projects are two general-initiative virtual labs that offer both a remote framework and a broader scope (Potkonjak et al., 2016). The Go-Lab project is a large collection of interactive virtual labs that enables teachers to develop inquiry learning spaces by combining online laboratories and applications. The learning space can be shared with teachers and students for creating and testing hypotheses as well as designing educational games (Dziabenko & Budnyk, 2019). The “Library of Labs (LiLa)” project creates an infrastructure for virtual experimentation. It goes beyond just gaining scientific knowledge by offering social communication skills with colleagues and mentors (Richter et al., 2011). Various commercial software packages are recently available with immersive simulators, for example, Labster, which has multiple virtual labs in different disciplines with game-based components to motivate students to learn techniques, solve problems, and apply experiments. Labster and other comparable programs like Late Nite Late Labs let students actually feel as if they are in the lab through the simulation environment to improve the immersion quality (Jones, 2018).

Virtual labs provide students with a personalized immersive learning experience through immersive tools such as virtual reality (VR), augmented reality (AR), and mixed reality (MR) for use within education (Hauze & Frazee, 2019). Early research suggests that immersive simulation improves student skills, knowledge, and motivation to learn (Chiu et al., 2015; Freina & Ott, 2015; Salmi et al., 2017; Zhang et al., 2014). VR has been widely used in a variety of educational settings. High school students used VR in 3D interactive chemistry labs (Ali et al., 2014; Civelek et al., 2014). Many articles focus on higher education; for instance, students in computer science courses have tested VR as an intelligent learning environment (Griol et al., 2014). A VR immersive environment can be used to design architectural spatial experiences (Ângulo & Velasco, 2014) and the presentation of neutrino data (Izatt et al., 2014). VR has been widely utilized in the field of medical education, particularly for applications such as nurse education in an interactive virtual environment (Green et al., 2014), simulated hospitals in medical education (Kleven & Prasolova-Førland, 2014), a caries removal simulation for dental students (Eve et al., 2014), and finger tracking using a head-mounted display to show surgeons how the expert's fingers move during surgery. Furthermore, VR is utilized directly with patients for educational purposes (de Ribaupierre et al., 2014; Rodrigues et al., 2014).

Many other virtual labs were developed as discipline-based labs, such as the Open-Source Physics (OSP) project improves computational physics education by providing simulators for basic techniques as well as education (Christian et al., 2011). In engineering, the TriLab project, which includes three access modes “hands-on, virtual, and remote lab” provides students with control engineering concepts and loop control using “Laboratory Virtual Instrument Engineering Workbench (LabVIEW)” (Abdulwahed & Nagy, 2013). In biology, the BioInteractive provides classroom resources and improves biology teachers’ content with scientific-based multimedia resources and stories to motivate students (Beardsley et al., 2022). In chemistry, ChemCollective involves virtual labs, educational materials as alternatives to textbooks, and student- or team-based activities (Yaron et al., 2010). The students can work with hundreds of chemicals and manipulate them without extra cost or possible risks (Yaron et al., 2010). According to a literature review on chemical virtual labs, there is a limitation in updating virtual labs based on student level, and the information provided by current virtual laboratories is static and limited in analytics (Ali & Ullah, 2020).

### Learning analytics

Educational technology has evolved in three distinct waves. The first wave started with the development of learning management systems (LMS). Social networks are considered the second wave of educational development that affects learning. Learning analytics, which is the third wave, is used to improve and optimize education (Fiaidhi, 2014). LA as a multidisciplinary field has been drawn from diverse scientific fields including computer science, education science, data mining, statistics, pedagogy, and behavioral science (Chatti et al., 2012).

The main objectives that have been explored in LA research are to support instructional strategies and the most promising applications in education, identify at-risk students to provide effective interventions; recommend reading materials and learning activities to students; and assess their outcomes (Romero & Ventura, 2020). The use of LA allows for tracking students' activities and providing feedback to improve the learning experience. LA pursued its objectives using various data mining techniques to create analytical models, which give a deep look into the learning process and could lead to more effective learning and pedagogical intervention (Elmoazen et al., 2022; Heikkinen et al., 2022). Among the approaches utilized, improved, or introduced in LA are machine learning, predictive analytics, process and sequence mining, and social network analysis (Romero & Ventura, 2020). The initial work was mostly algorithms for the prediction of students' success, and at-risk student identification (Ifenthaler & Yau, 2020). Then some researchers argued that relying on learning analytics for prediction is not sufficient (Saqr et al., ; Tempelaar et al., 2018), and it is essential to include pedagogical perspectives while studying the learning process (Gašević et al., 2015; Wong et al., 2019). Accordingly, scholars give more attention to pedagogical practices and feedback in recent LA research (Banihashem et al., 2022; Wise & Jung, 2019).

In virtual labs, LA techniques were applied in a variety of approaches to investigate the impact of using virtual labs to gain the necessary skills and competencies. Govaerts et al. (2012) applied the Student Activity Meter (SAM) to visualize students' performance based on many metrics, which they then displayed in a comprehensive dashboard with dimensional filtering. Similarly, in the FORGE European online learning project, a dashboard was used to visualize students' interactions with course materials and each other, in addition to surveys and questionnaires (Mikroyannidis et al., 2015). The dashboards of virtual labs present a summary of student progress by visualization using different statistical charts such as histograms and plots (Garcia-Zubia et al., 2019; Tobarra et al., 2014).

Many research papers use interaction data, including statistical extraction of students' interactions in relation to time spent, the distribution of time-on-task per student, and different user configurations (Elmoazen et al., 2022; Heikkinen et al., 2022; Ifenthaler & Yau, 2020). Another approach is to develop an autonomous assessment and recommendation system to analyze real-time activity results and improve students’ performance in virtual labs (Considine et al., 2019; Gonçalves et al., 2018). For instance, for optimal performance of virtual labs, students should spend appropriate amounts of time interacting with tools and resources. The relationship between students' interactions and their academic progress may be used to study students' behavior. Moreover, clustering methodologies can categorize students by their weaknesses and strengths to study their learning progress (Tulha et al., 2022).

## Methodology

The authors conducted this review according to the Preferred Reporting Items for Systematic Reviews and Meta-Analyses (PRISMA) 2020 guidelines (Page et al., 2021) and the eight fundamental steps of systematic reviews by Okoli (2015). The authors followed these guidelines to identify the purpose of the review, prepare a protocol draft, identify inclusion and exclusion criteria, and conduct the search process in order to extract data and appraise articles' quality before writing the review.

First, the authors determined that the purpose of the study was to report on the application of learning analytics in virtual labs to answer research questions. Following the assessment of the review's scope, the authors frequently convened to draft the protocol. This document organizes all subsequent actions to reduce the possibility of bias in the selection of publications and data processing. The protocol ensures reproducibility and consistency by planning the strategy for practicing and conducting the review (Fink, 2019). Accordingly, the protocol included research questions, the literature search strategy, inclusion criteria, the assessment of the studies, the data extraction, and the planned schedule (Kitchenham & Charters, 2007).

The inclusion and exclusion criteria for study selection were based on the research questions and guided by ) previous review. All reviewed articles to be included should use learning analytics in virtual labs and meet the following inclusion and exclusion criteria:

1. Publications are written in English.
2. Journal articles, conference proceedings, and book chapters in their entirety. Thus, we excluded editorials, conference abstracts, workshop proposals, and posters.
3. Empirical studies with empirical data collection and analysis. Reviews and incomplete reports (e.g., abstract-only papers or papers without methods and results) were excluded.

### Database and literature search

The authors identified three established databases for the search: Scopus, Web of Science (WoS), and ERIC. Both Scopus and WoS databases employ rigorous inclusion criteria for journals and conferences, have a robust meta-data system, and have been established as literature search venues (Kumpulainen & Seppänen, 2022). ERIC is an educational database that covers a wide range of educational literature (Robbins, 2001). Additionally, the same keywords were used to search in the database of the Journal of Learning Analytics, the official publishing outlet for learning analytics.

We performed several iterations of search using different combinations of keywords; using the keyword “virtual” severely limited our findings and missed several papers. Some of the authors of the papers used other keywords, e.g., online laboratories, or did not use the keyword "virtual" at all within their keywords and therefore were not captured by the initial keywords that included "virtual." Therefore, a decision was made to cast a wide net, and retrieve any article that includes the keyword “lab*” with a wild card and then qualitatively—by the expert eyes of researchers—identify which of such keywords’ articles are about virtual laboratories. After several iterations, the following search formula yielded the best results for capturing all forms of keywords (Table 1):

( "learning analytics") AND ( lab* OR experiment* OR clinic* OR practical* OR immers*))

**Table 1:** The used keywords with wildcards to cover all keyword forms

Lab*	Lab, labs, laboratories, laboratory
Experiment*	*Experiment, experiments, experimental, experimentation*
Clinic*	Clinic, clinics, clinical, clinically, clinician
Practical*	Practical, practicals, practically
Immers*	Immerse, immersing, immersive, immersible

This combination of keywords was selected to be searched in the fields of the title, abstract, or author keywords of articles. The search was conducted within two days of the eighth of November 2021. The returned search resulted in 1069 articles from all specified databases, as follows: 653 articles from Scopus, 248 articles from WoS, 120 articles from the ERIC database, and 48 articles from the Journal of Learning Analytics. All articles were uploaded to the Covidence web-based system^1^ for analysis. Duplicates (n = 280) were removed, resulting in 789 articles. Two researchers independently scanned and assessed the first 100 papers' abstracts, titles, and keywords. The inter-rater agreement showed strong reliability using Cohen's Kappa test (κ = 0.92), and any conflicts were discussed and resolved, i.e., when the two authors had differing views about the classification of the paper, they discussed it until they reached a consensus.

**Fig. 1:**
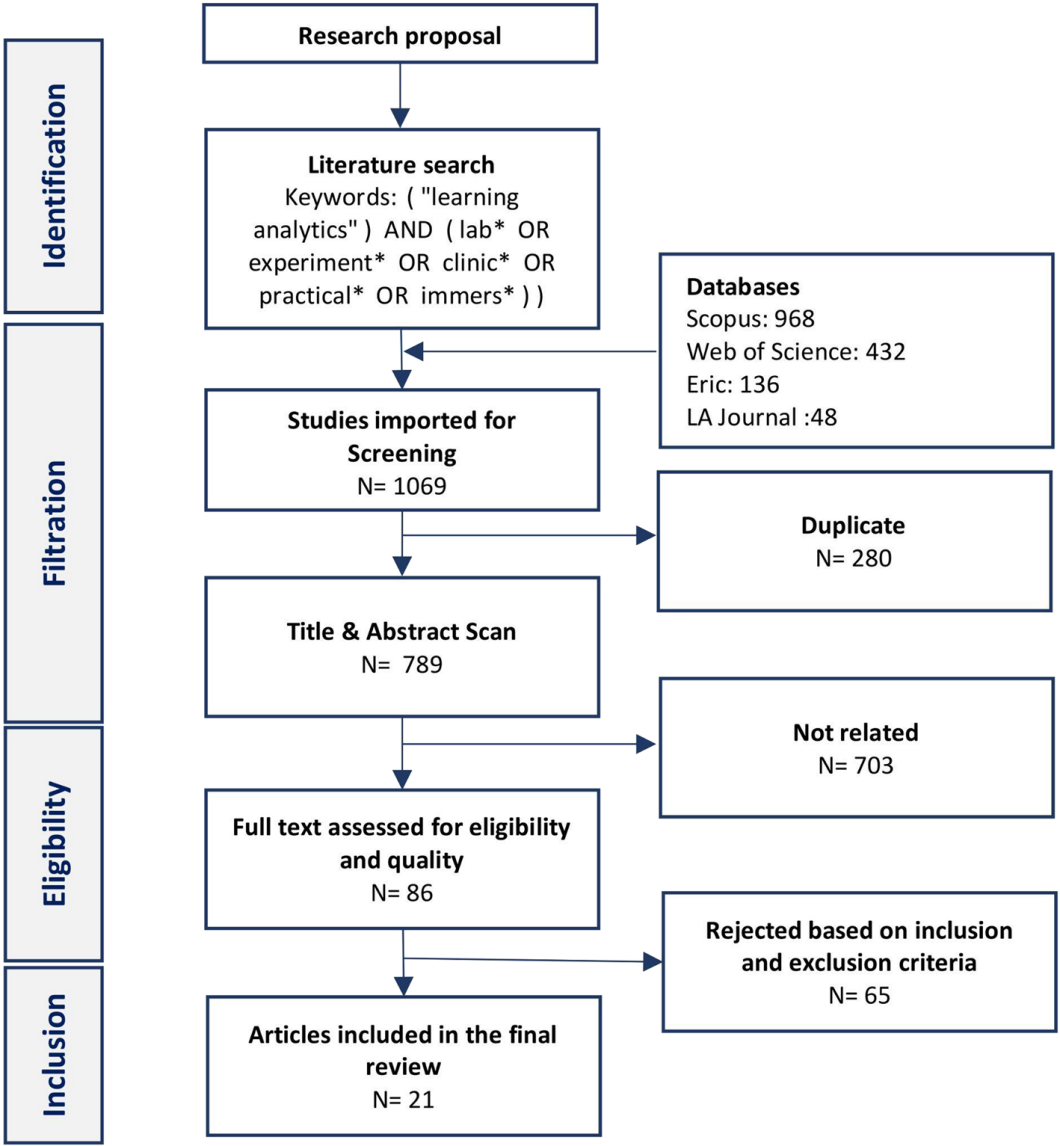
The study selection process

The remaining articles were divided and filtered by both researchers. All authors met after filtration to discuss any uncertainties. Based on the inclusion and exclusion criteria, the title and abstract scan yielded 86 publications that were suitable for full-text review (Fig. 1).

In order to obtain data from the included articles, the relevant information was first collected in a codebook. This was done to reduce the individual differences that existed between the reviewers. The following categories of information were extracted from each article: descriptive statistics, educational settings and levels, disciplines, learning analytics approaches, and the primary conclusions of each study. The first ten studies were coded by two different coders, and then they had a meeting to discuss any conflicts and complete the codebook before continuing to code articles. Finally, the retrieved papers were checked for quality before beginning the stage of synthesis. At this point, the writers are organizing all of the data within the framework of the review hypothesis (Webster & Watson, 2002). The data analysis gets a comprehensive presentation from a learning analytics perspective.

## Results

The included studies are listed in the appendix, and each one is given a capital **S** and a number.

### Descriptive statistics of the reviewed articles

**Fig. 2:**
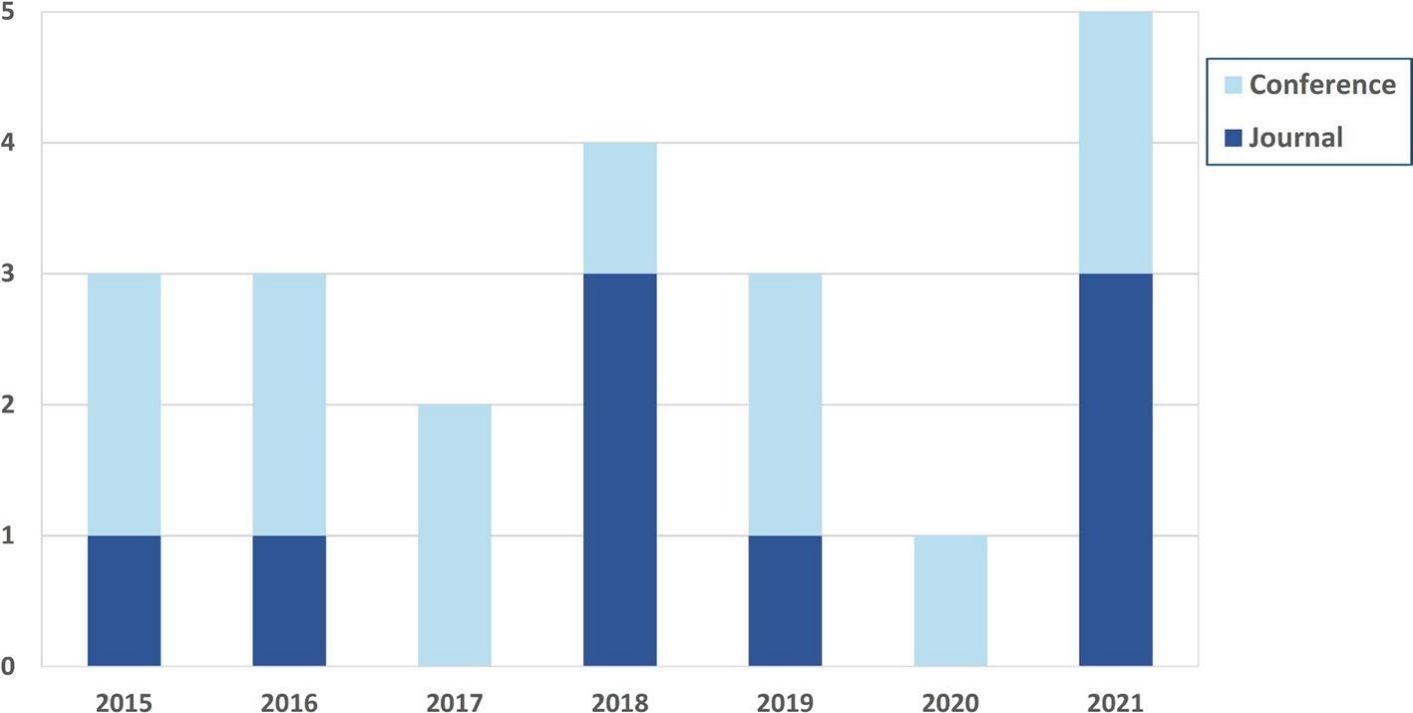
Type and year of the reviewed articles

There are a total of 21 studies that have been incorporated into this review. Before the year 2015, there were no studies utilizing learning analytics in virtual labs. All articles were published between 2015 and 2021. The maximum number of studies per year was five articles in 2021 followed by four articles in 2018. The majority of the reviewed articles were presented at conferences (N = 12), whereas the remaining nine articles were published in journals (Fig. 2).

### Educational levels

**Fig. 3:**
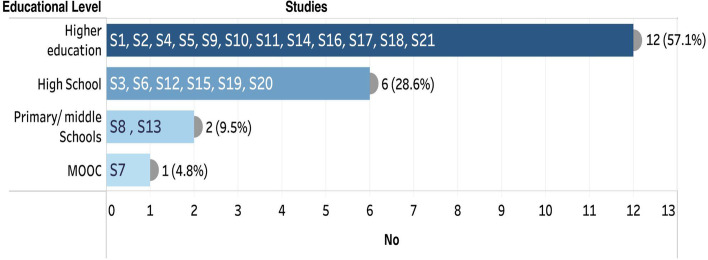
Educational Levels in the reviewed studies

The reviewed studies have populations from various educational levels (Fig. 3). The majority of the research in the reviewed articles (57.1%) was conducted in higher education institutions (n = 12) and two of these studies involved postgraduate students in their analysis (Burbano & Soler, 2020; Considine et al., 2021). Six studies (28.6%) were conducted on secondary education, and four of them focused on STEM (science, technology, engineering, and math) subjects (de Jong et al., 2021; Rodríguez-Triana et al., 2021; Sergis et al., 2019; Vozniuk et al., 2015). Only two research projects (9.5%) focused on elementary and middle school students (Metcalf et al., 2017; Reilly & Dede, 2019a). Finally, one study was conducted online as a Massive Open Online Course (MOOC) for students of varying education levels (Hossain et al., 2018).

### Subjects

**Fig. 4:**
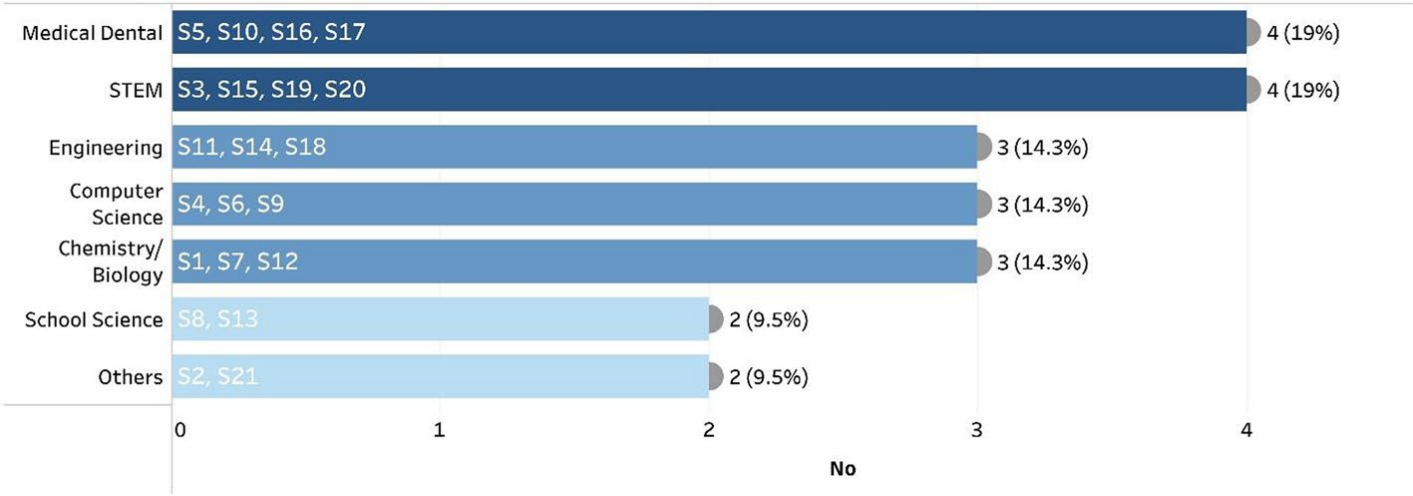
Context of the reviewed studies

The reviewed studies covered different disciplines of science, medicine, and engineering (Fig. 4). The medical and dental virtual practices were used in practical-based physiology courses (King et al., 2016), virtual patient cases (Berman et al., 2018), periodontology and oral pathology (Burbano & Soler, 2020) and prosthodontics courses (Chan et al., 2021a, 2021b). Chemistry virtual labs were used in concentration experiments (Liu et al., 2018) and organic chemistry (Qvist et al., 2015), while biology labs covered Euglena’s interactive live (Hossain et al., 2018), and molecular biology experiments (Qvist et al., 2015). Virtual labs for science classes were available for school students (Metcalf et al., 2017; Reilly & Dede, 2019b) and students in science, technology, engineering, and mathematics (STEM) (de Jong et al., 2021; Rodríguez-Triana et al., 2021; Sergis et al., 2019; Vozniuk et al., 2015). Virtual labs were used in different fields of computer science, namely Java programming (Castillo, 2016), cloud applications (Manske & Hoppe, 2016), and network virtual labs (Venant et al., 2017). The engineering virtual labs covered automotive engineering (Goncalves et al., 2018), container-based virtual labs (Robles-Gómez et al., 2019), and building electrical circuits (Considine et al., 2021). Other practices include digital electronic simulation environments (Considine et al., 2021) and remote labs in the field of image processing (Vahdat et al., 2015).

### Virtual environment

The authors of the reviewed articles used a wide range of virtual environments. Go-lab was used in STEM education (de Jong et al., 2021; Rodríguez-Triana et al., 2021; Sergis et al., 2019) and was combined with other applications such as the GRAASP platform (Vozniuk et al., 2015) and cloud applications (Manske & Hoppe, 2016). In addition, the EcoXPT system was utilized in science classes. (Metcalf et al., 2017; Reilly & Dede, 2019b). In the medical field, the LabTutor platform was used in physiology courses (King et al., 2016), the ASUS virtual patient package (Berman et al., 2018), and the M-Health Smilearning application with TIMONEL platform in the dental field (Burbano & Soler, 2020). Chemistry virtual labs were accessible on two platforms: the ChemVLab + tutor (Liu et al., 2018) and the LabLife3D platform (Qvist et al., 2015). In the field of biology, virtual labs were available in LabLife3D for molecular biology (Qvist et al., 2015) and Open edX for Euglena experiments (Hossain et al., 2018). The virtual labs used in computer science were the Magentix 2 platform with virtual hosts (Castillo, 2016), and the network Lab4CE (Laboratory for Computer Education) (Venant et al., 2017). Various engineering fields utilized different virtual lab platforms, such as Falstadat’s Circuit Simulator Applet and Virtual Instrumentation Systems in Reality (VISIR) (Goncalves et al., 2018), Netlab for building electrical circuits (Considine et al., 2021), and a container-based virtual laboratory (CVL) using Linux Docker containers (Robles-Gómez et al., 2019). Other labs included such as DEEDS (Digital Electronics Education and Design Suite) for digital electronic simulation environments (Vahdat et al., 2015) and the WebLab-Deusto remote lab management system (RLMS) for image processing (Schwandt et al., 2021).

### Perception of virtual labs

The findings reported that virtual labs are inexpensive, robust (Hossain et al., 2018), and have a very high satisfaction level among students (Castillo, 2016). Students recorded their positive feedback and interest in virtual labs as they simplified complex scientific practices (Hossain et al., 2018; Qvist et al., 2015; Robles-Gómez et al., 2019). Similarly, some post-graduate students preferred remote labs after their experience during the COVID-19 pandemic (Considine et al., 2021). Regarding the teachers, they displayed a positive response regarding learning analytics in virtual labs as they can monitor students’ progress (Qvist et al., 2015; Vozniuk et al., 2015). The teachers expressed the need for an enhancement in displaying students’ activities and technical guidelines to support inquiry-based learning in virtual labs (Rodríguez-Triana et al., 2021). Many authors showed evidence of improvement in students’ performance with the use of virtual labs (King et al., 2016; Manske & Hoppe, 2016; Metcalf et al., 2017; Robles-Gómez et al., 2019).

### Learning analytics

The reviewed studies mainly covered one or more of these variables: performance, activities, perception, and behavior. Performance was assessed in 11 studies, either to evaluate the impact of the virtual labs on learning achievement (King et al., 2016; Metcalf et al., 2017; Reilly & Dede, 2019b; Robles-Gómez et al., 2019; Vahdat et al., 2015); improve knowledge (Burbano G & Soler, 2020; Manske & Hoppe, 2016); assess the need for support (Goncalves et al., 2018; Venant et al., 2017) or assess the inquiry-based educational designs by teachers (de Jong et al., 2021; Sergis et al., 2019). There are 10 studies focusing on the analysis of students’ activities and the pattern of virtual lab utilization (Castillo, 2016; King et al., 2016; Hossain et al., 2018; Metcalf et al., 2017; Berman et al., 2018; Liu et al., 2018; Burbano and Soler 2020; Considine et al., 2021; Schwandt et al., 2021; Chan et al., 2021a, 2021b). Nine studies measured the perceptions towards virtual labs in one of three forms: self-reported feedback (Berman et al., 2018; Chan et al., 2021a, 2021b; Considine et al., 2021; Hossain et al., 2018), teacher’s opinions (Qvist et al., 2015; Rodríguez-Triana et al., 2021; Vozniuk et al., 2015) and students’ satisfaction questionnaires (Castillo, 2016; Robles-Gómez et al., 2019). Three studies identified the behavior pattern of the students in virtual labs (Robles-Gómez et al., 2019; Vahdat et al., 2015; Venant et al., 2017).

The learning analytics in the reviewed articles were mainly based on log data from the virtual lab platforms. The data collected from system log files consist of general data such as user ids, students’ clicks, the start and end of experiments, and users’ actions (Schwandt et al., 2021). Authors used log files to analyze the patterns of experiments (Metcalf et al., 2017; Qvist et al., 2015), long-term patterns in MOOC courses (Hossain et al., 2018), and interactions between learners (Venant et al., 2017). Some authors used the time sequence as part of their analysis to monitor the timeline pattern (Qvist et al., 2015), durations of system activities (Burbano G & Soler, 2020; Vozniuk et al., 2015), time spent on tasks (King et al., 2016), sequence of actions (Manske & Hoppe, 2016) and comparison between more than one academic year to assess the improvement when using virtual labs (Robles-Gómez et al., 2019). Also, the students’ performance can be predicted using engagement metrics of student activity (Berman et al., 2018; Castillo, 2016), complexity metrics (Vahdat et al., 2015), and behavior during practical learning (Venant et al., 2017). Thus, learning analytics help teachers figure out when students are having difficulties and support them when needed (Goncalves et al., 2018; Sergis et al., 2019; Venant et al., 2017).

Process mining was used as a temporal method to discover the hidden strategies of students to achieve their goals (Castillo, 2016). Similarly, students’ learning strategies and practical activity sequences were analyzed using sequential pattern mining to identify behavior variations at different performance levels (Venant et al., 2017). The learning trajectories of students were identified by meta-classification of the events with their timestamps (Reilly & Dede, 2019b) and by selecting the segments of interest in log data and then coding the video and audio recordings for these segments (Liu et al., 2018). Correlation analysis and multiple linear regression analysis were used to address the relationship between access to learning resources and academic achievement (Chan et al., 2021a, 2021b). Students’ performance was part of the analysis by monitoring the students’ results in exams (Goncalves et al., 2018) and extracting their mistakes (Considine et al., 2021). Virtual labs included built-in learning analytics tools in many studies such as the Learning Analytics Data Collector (LADC) (Vahdat et al., 2015), Inquiry Learning Space (ILS) dashboard, “Teaching and Learning Analytics (TLA)” and measurements based on algorithms to analyze the correlation between students’ performance and actions (Schwandt et al., 2021). Finally, virtual patients’ metrics were used to monitor students (Berman et al., 2018).

## Discussions and conclusions

This study is aimed at reviewing research at the intersection of learning analytics and virtual laboratories. While learning analytics emerged more than a decade ago, the number of articles that particularly focus on virtual laboratories remains paltry, and the growth curve is largely flat with a yearly frequency of three to four articles per year. The included articles (n = 21) were published in the last six years, pointing to a rather cautious adoption trend among educators. In fact, systematic literature reviews have pointed to a slow adoption trend within scientific education fields with the faint appearance of articles from these domains, e.g., (Ifenthaler & Yau, 2020; Saqr, 2018). The results came from diverse fields with a concentration around STEM, health sciences (Burbano G and Soler 2020; Chan et al., 2021a, 2021b), science (chemistry and biology), as well as engineering sciences (Considine et al., 2021; Goncalves et al., 2018; Robles-Gómez et al., 2019). There was a diverse repertoire of digital platforms; almost every study used a different platform. Such a wide diversity across contexts and digital platforms is giving rise to fragmentation of the insights, making it hard to draw a consistent conclusion or a common narrative. In other words, since most of the experimental findings come from a different context with a specific platform, we can hardly reach a conclusion that applies in other cases that do not use such a platform or come from a different platform.

The reported results—by the reviewed papers—have studied students’ perceptions of the virtual laboratories (Berman et al., 2018; Chan et al., 2021a, 2021b; Hossain et al., 2018), performance, and online behavior (e.g., using log data). Obviously, students’ perceptions or performance are not well related to learning analytics, yet they continue to receive researchers’ attention. In particular, the issue of improving performance has witnessed rising adoption in the last decade as stakeholders wish to use data to improve students’ learning. Log data within the reviewed studies formed the basis of most analyses and revolved around understanding behavioral patterns of using online laboratories, or how using such platforms can help us predict or understand students’ performance. Less frequently, studies have tried to map students’ temporal behavioral patterns using e.g., sequence (Such studies—that used temporal methods—offered valuable insights about students’ laboratory learning strategies and the sequence of virtual lab activities). Some of the reviewed studies had built-in analytics solutions in the form of dashboards specific to such platforms. Teachers’ perspectives have been investigated in several studies, in which they reported a positive perception of the potential of learning analytics, e.g., enabling students’ monitoring, helping support students’ during laboratory work, and offering ways for scaffolding.

The small number of studies in this review, which are distributed across different fields, platforms, and methods, makes it hard to draw any general conclusions. It is, therefore, fair to say that studies hitherto are still in an exploratory stage. Several areas of research and questions are still unanswered, e.g., what are the effective strategies when using online laboratories, what are the indicators that point to a student needing help; what are the effective supportive strategies, and what are the indicators that best predict that a student is benefiting from online laboratories. What is more, we have little information about interactivity in virtual labs, their patterns, benefits, or lack thereof, and how to best support such strategies. In addition, we stress the findings by Birkeland (Birkeland, 2022) regarding the absence of collaborative environments and teamwork incentives, which are very common practices in virtual labs, pointing to a critical issue that current virtual labs lack.

Since studies do not use a common standard, protocol, or shared methods, their findings are not shared, portable, or built on each other. Authors and researchers need to think of common protocols, standards, or application programming interfaces (APIs). Such common protocols would make efforts more likely to build on each other and results more likely to be shared.

Virtual laboratory dashboards are in the very early stages of development, and little is known about the effective elements of dashboards that could help students or teachers. In fact, how learning analytics can help teachers optimize learning and teaching with virtual laboratories is still an open area of inquiry. In the same vein, how learning analytics can help teachers design, assess, or improve learning tasks is still largely unexplored.

This systematic review comes with the following limitations. The search was performed using five search terms in the title and keywords, which were too generic and resulted in a large number of articles being initially reported and then excluded. This may complicate the search and filtration processes, but it reduces the exclusion of any articles, as authors didn't include "virtual labs" in their keywords. However, if authors didn’t include our keywords, their work may have been missed in this review. Although the coding process for this review worked well for most articles, the coders had to make an effort to interpret some articles. Thus, in order to facilitate coding, the authors had to discuss and figure out the primary emphasis of the research that was unclear. Also, construct validity may be needed as we rely on author descriptions and code groupings that sometimes differ from the author's domains, which don’t follow a standardized approach or protocol. Finally, the qualitative analysis and the relatively small number of articles included in this review from a variety of disciplines and research approaches restrict the ability to make broad generalizations due to a lack of standardization”. Nonetheless, this research presents the first systematic overview of learning analytics in virtual labs. Researchers may utilize our work as a framework and lens to perform further rigorous research, and we believe that the results we have provided can serve as a new basis for learning analytics in laboratories.

In summary, our review addressed questions pertaining to the use of learning analytics in virtual laboratories. An area that still has significant gaps of knowledge that only future research would help us shed light on.

## Data Availability

The data of this systematic review consist of articles published in journals and conferences. Many of these are freely available online, others can be accessed for a fee or through subscription.

## Appendix

**Table:**

No	Study ID	Title	Aim of study/research question
S1	Qvist 2015 (Qvist et al., 2015)	Design of Virtual Learning Environments Learning Analytics and Identification of Affordances and Barriers	To present the design and implementation of virtual laboratories, and to discover student and teacher views on the affordances and barriers to learning in these environments
S2	Vahdat 2015 (Vahdat et al., 2015)	A learning analytics approach to correlate the academic achievements of students with interaction data from an educational simulator	Understanding the learning behavior of students while interacting with Technology Enhanced Learning (TEL) systems
S3	Vozniuk 2015 (Vozniuk et al., 2015)	Contextual learning analytics apps to create awareness in blended inquiry learning	RQ1. Do such contextual real-time visualisations improve teacher’s awareness? RQ2. Are the apps understandable and easy to use?
S4	Castillo, 2016 (Castillo, 2016)	A virtual laboratory for multiagent systems: Joining efficacy, learning analytics and student satisfaction	It aims at capturing the daily activity of students, providing the basis for data-driven assessment, and introducing a distributed virtual laboratory for a multiagent programming course
S5	King 2016 (King et al., 2016)	Evaluation and use of an online data acquisition and content platform for physiology practicals and tutorials	1) to examine the usage pattern of students during delivery of one module of the online practical courseware, “Electrophysiology of the Nerve”, over the first two years of its implementation 2) to gather evidence of the impact of the platform on student engagement and learning outcomes
S6	Manske 2016 (Manske & Hoppe, 2016)	The "Concept cloud": Supporting collaborative knowledge construction based on semantic extraction from learner-generated artefacts	Propose the use of computational methods of semantic extraction to better understand and reflect on the activities in the Go-Lab online learning environment
S7	Hossain 2018 (Hossain et al., 2018)	Design Guidelines and Empirical Case Study for Scaling Authentic Inquiry-based Science Learning via Open Online Courses and Interactive Biology Cloud Labs	To demonstrate that the cloud lab technology in question can support authentic science inquiry-based learning at large scale, and to distill design principles from the core technology, the user interface, and the course for successful deployments of online labs and courses for inquiry-based learning
S8	Metcalf 2017 (Metcalf et al., 2017)	Changes in Student Experimentation Strategies within an Inquiry-Based Immersive Virtual Environment	1. How did students use the Mesocosm tool over time? Did their patterns of use change over time, in terms of number of pools, number of measurements collected, and use of a control? 2. How did students interpret Mesocosm experimental results over time? Was there a change in students' connection of experimental results and their conceptual understanding of the causal relationships affecting the ecosystem?
S9	Venant 2017 (Venant et al., 2017)	Using sequential pattern mining to explore learners’ behaviors and evaluate their correlation with performance in inquiry-based learning	The objective is to identify behavioural patterns for a practical session that lead to better learning outcomes, to predict learners’ performance and to automatically guide students who might need more support to complete their tasks
S10	Berman 2018 (Berman et al., 2018)	Development and initial validation of an online engagement metric using virtual patients	Do student actions while completing an online virtual patient case reflect their engagement?
S11	Goncalves 2018 (Goncalves et al., 2018)	Personalized student assessment based on learning analytics and recommender systems	Analysis of student assessment to provide clues to help teachers in scaffolding the students' performance
S12	Liu 2018 (Liu et al., 2018)	A Novel Method for the In-Depth Multimodal Analysis of Student Learning Trajectories in Intelligent Tutoring Systems	Describing a generalizable approach for combining quantitative and qualitative analyses to yield efficient yet rich sensemaking around intelligent tutoring data
S13	Reilly 2019 (Reilly & Dede, 2019b)	Differences in student trajectories via filtered time series analysis in an immersive virtual world	This study aims to explore ways time-stamped log files of groups’ actions may enable the automatic generation of formative supports
S14	Robles-Gómez 2019 (Robles-Gómez et al., 2019)	Analyzing the students' learning within a container-based virtual laboratory for cybersecurity	This work focuses on the proposal and analysis of a container-based virtual laboratory for a "cybersecurity" subject, from the point of view of the students’ behavior and their outcomes
S15	Sergis 2019 (Sergis et al., 2019)	Using educational data from teaching and learning to inform teachers’ reflective educational design in inquiry-based STEM education	Investigates whether Teaching Analytics can be used to assess Inquiry-based Educational Designs (IED) and relate analyses to customizable students' educational data to facilitate the re-design process
S16	Burbano 2020 (Burbano G & Soler, 2020)	Learning analytics in m-learning: Periodontic education	Understand the transformation of educational and training systems from the perspective of the ubiquitous learning experience of medical and dental students
S17	Chan 2021 (Chan et al., 2021a, 2021b)	The relation of online learning analytics, approaches to learning and academic achievement in a clinical skills course	1. effect of students approach on access of e-learning resource 2. effect of students’ approaches to learning and access of e-learning on academic achievement examination results
S18	Considine 2021 (Considine et al., 2021)	An Automated Support System in a Remote Laboratory in the Context of Online Learning	Reports on learning habits of the students, their backgrounds and their perception of online learning preceding and following the use of the automated tutoring system
S19	DeJong 2021 (de Jong et al., 2021)	Understanding teacher design practices for digital inquiry-based science learning: the case of Go-Lab	Analyze how teachers design Inquiry Learning Spaces (ILSs) for online learning with STEM-related online laboratories in Go-Labs
S20	Rodríguez-Triana 2021 (Rodríguez-Triana et al., 2021)	ADA for IBL: Lessons learned in aligning learning design and analytics for inquiry-based learning orchestration	1) What are the orchestration needs of teachers implementing IBL in their classrooms? 2) To what extent do "alignment of design and analytic" (ADA) solutions fulfill such orchestration needs?
S21	Schwandt 2021(Schwandt et al., 2021)	Utilizing User Activity and System Response for Learning Analytics in a Remote Lab	Perform learning analytics by recording the user interactions and the behavior inside the remote lab

## Footnotes

1 https://www.covidence.org/ (last accessed September 2022).
